# Analysis of variable major protein antigenic variation in the relapsing fever spirochete, *Borrelia miyamotoi*, in response to polyclonal antibody selection pressure

**DOI:** 10.1371/journal.pone.0281942

**Published:** 2023-02-24

**Authors:** Robert D. Gilmore, Brittany A. Armstrong, Kevin S. Brandt, Taylor J. Van Gundy, Andrias Hojgaard, Job E. Lopez, Alexander R. Kneubehl

**Affiliations:** 1 Bacterial Diseases Branch, Division of Vector-Borne Diseases, National Center for Emerging and Zoonotic Infectious Diseases, Centers for Disease Control and Prevention, Fort Collins, Colorado, United States of America; 2 Department of Molecular Virology and Microbiology, Baylor College of Medicine, Houston, Texas, United States of America; 3 Department of Pediatrics, National School of Tropical Medicine, Baylor College of Medicine, Houston, Texas, United States of America; University of Kentucky College of Medicine, UNITED STATES

## Abstract

*Borrelia miyamotoi* is a tick-transmitted spirochete that is genetically grouped with relapsing fever *Borrelia* and possesses multiple archived pseudogenes that encode variable major proteins (Vmps). Vmps are divided into two groups based on molecular size; variable large proteins (Vlps) and variable small proteins (Vsps). Relapsing fever *Borrelia* undergo Vmp gene conversion at a single expression locus to generate new serotypes by antigenic switching which is the basis for immune evasion that causes relapsing fever in patients. This study focused on *B*. *miyamotoi vmp* expression when spirochetes were subjected to antibody killing selection pressure. We incubated a low passage parent strain with mouse anti-*B*. *miyamotoi* polyclonal antiserum which killed the majority population, however, antibody-resistant reisolates were recovered. PCR analysis of the gene expression locus in the reisolates showed *vsp1* was replaced by Vlp-encoded genes. Gel electrophoresis protein profiles and immunoblots of the reisolates revealed additional Vlps indicating that new serotype populations were selected by antibody pressure. Sequencing of amplicons from the expression locus of the reisolates confirmed the presence of a predominant majority serotype population with minority variants. These findings confirm previous work demonstrating gene conversion in *B*. *miyamotoi* and that multiple serotype populations expressing different *vmps* arise when subjected to antibody selection. The findings also provide evidence for spontaneous serotype variation emerging from culture growth in the absence of antibody pressure. Validation and determination of the type, number, and frequency of serotype variants that arise during animal infections await further investigations.

## Introduction

*Borrelia miyamotoi* is an emerging tick-borne bacterial pathogen first discovered from *Ixodes persulcatus* ticks in Japan in 1994 but was not described as a human pathogen until 2011 where the spirochete was isolated from patients in Russia with subsequent clinical identifications in North America and Europe [1–3]. *B*. *miyamotoi* is phylogenetically related to the Relapsing Fever (RF) group of borrelial spirochetes that includes *B*. *hermsii*, *B*. *turicatae*, and *B*. *parkeri* among others. However, unlike these RF organisms, *B*. *miyamotoi* is not transmitted by *Ornithodoros* spp. ticks, but rather by *Ixodes* spp. ticks, the arthropod vector that harbors and transmits the Lyme disease spirochete, *Borrelia (Borreliella) burgdorferi* [1–4].

RF *Borrelia* infection of humans, e.g., by *B*. *hermsii*, results in a series of febrile episodes between periods of recovery due to antigenic variation by the invading spirochete. This intricate immune evasion strategy creates new serotype populations that evade host antibodies elicited by the initial infecting borreliae [5, 6]. Host antibodies are directed against a surface protein that determines serotype in the infecting population [7–9]. These immunogenic surface proteins are termed variable major proteins (Vmps) and are encoded by archived plasmid-localized pseudogenes that will recombine into a single expression locus [10–15]. Antigenic variation occurs by a gene conversion event whereby a transcriptionally inactive pseudogene is translocated into the active expression site resulting in a new serotype population [5, 10, 16]. Although only a single Vmp is expressed at one time in an organism, mixed populations can coexist that display different Vmp types. The Vmps are separated into two groups by molecular size: approximate 25 kDa proteins termed variable small proteins (Vsps) and larger approximate 37 kDa proteins termed variable large proteins (Vlps). Vlps are subdivided into alpha, beta, delta, and gamma subfamilies [14]. Vmp-like genes in *B*. *miyamotoi* with homology to *B*. *hermsii vmps* was first reported by Hamase et al and confirmed by DNA sequencing in a study by Barbour [17, 18]. *Borrelia* spp. genomes consist of an approximate megabase chromosome with linear and circular plasmids that can differ in numbers and sizes in strains and isolates. *B*. *miyamotoi* genome sequences are available for strains isolated from North America, Japan, Russia, and the Netherlands and show that *B*. *miyamotoi* possesses a collection of plasmid-localized Vmp pseudogenes similar to those of *B*. *hermsii* and *B*. *turicatae* (https://www.ncbi.nlm.nih.gov/genome/genomes/16651 and https://www.ncbi.nlm.nih.gov/genome/plasmids/16651).

A single expression locus with an active promoter for *vsp1* gene expression was described in a genetic analysis of *B*. *miyamotoi* strain LB-2001 plasmid-localized *vmps* [17]. Sequences showed nucleotide differences between the active promoter located on one plasmid and the silent upstream regions of a *vsp1* pseudogene paralog. Additionally, the silent *vsp1* differed from the expressed *vsp1* by a stop codon resulting from a frameshift just two codons downstream from the ATG start indicating the divergence between expressed and silent genes [17]. A study by Wagemakers et al detailed that a parent LB-2001 strain with *vsp1* in the expression locus converted into a new serotype (VlpC2) after being subjected to anti-Vsp1 antibodies thereby demonstrating antigenic variation by a gene conversion event in *B*. *miyamotoi* [19]. Outside of this finding by the Hovius laboratory, in depth studies into the mechanisms of antigenic variation in *B*. *miyamotoi* and subsequent comparisons to gene conversion events reported for *B*. *hermsii* and *B*. *turicatae* have yet to be investigated.

Our aim was to further characterize *B*. *miyamotoi* antigenic variation by gene conversion. To begin this investigation, we subjected *B*. *miyamotoi* strain LB-2001 to selection pressure with mouse polyclonal antiserum *in vitro* that would simulate the response encountered during mammalian infection. Our goal was to elicit cell death with borrelicidal antibodies and to assess whether reisolates with new serotype populations were generated, and if so, to identify *vmp* pseudogenes that were translocated into the expression locus. Here we show that Vmp-expressing variant populations were selected following pressure with polyclonal anti-*B*. *miyamotoi* antibodies and present evidence that new variants also spontaneously emerge by growth in culture.

## Materials and methods

### Strains and culture passage

*B*. *miyamotoi* strain LB-2001 was originally isolated from *I*. *scapularis* in the Northeast United States and was maintained by passage in SCID mice [20]. Low passage (≤4) *B*. *miyamotoi* strain LB-2001 was kindly supplied by Joppe Hovius, Center for Experimental and Molecular Medicine, Amsterdam, The Netherlands, and was subcultured to create low passage (<10) freezer stocks. *B*. *miyamotoi* strain CT13–2396 was isolated from an infected *I*. *scapularis* nymph collected in Connecticut, USA [21]. *B*. *miyamotoi* was cultivated in Modified Kelly-Pettenkofer Medium (MKP-F) [22] at 34°C in capped tubes and harvested at 4×10^7^ spirochetes/ml.

### PCR of expression site

DNA templates were prepared from 1 ml *B*. *miyamotoi* culture pellets (approximately 1 x 10^7^ cells) resuspended in sterile nanopure water (50 μl) and boiled for 5 min with 0.5–1 μl used in the PCR. PCRs were performed in 20 μl volume with 0.1 μM primer and 2X Dream Taq Green polymerase (ThermoFisher). Reaction parameters were 95° C for 2 min (1 cycle); 95° C for 30 sec, 55° C for 30 sec, 72° C for 60 sec (35 cycles); 72° C for 5 min (1 cycle). PCRs using the Vlp25R primer used a 50° C annealing temperature. Amplicons were run on 1% Tris-acetate-EDTA agarose gels. The expression site promoter forward primer, Pexp-F (ATAAAGAATTTGAAAAGTAAGATTCTTGCAC) was designed from the published sequence [17]. The Vlp25R reverse internal primer (CCACTATCAACTGTATTTCTTATTGCTA) was designed from a conserved region of 25 LB-2001 *vlp* genes after nucleotide sequence alignment using MegAlign Muscle algorithm (DNASTAR Lasergene, Madison, WI) approximately 1 kb into the coding sequence. The 25 Vlps were from subfamilies beta, delta, and gamma. The remaining 4 Vlps of subfamily alpha were aligned as above to design an internal reverse primer, Vlp4R, (CTTGCTTCATCTATTTTTTCTTTTGC). The VlpC2 reverse internal primer (CACCAGCATTCTGAGAAGTAGCTAATTTGGCA) was designed from the CT13-2396 strain GenBank sequence (AXH25_04655, annotated as variable large protein 5).

### Antibody killing/Selection assay

Mouse polyclonal anti-*B*. *miyamotoi* was generated as previously described by injecting CD-1 outbred mice with 1 x 10^5^ LB-2001 low passage (P4) [23]. *B*. *miyamotoi* strain LB-2001 low passage 8 (P8) was subjected to anti-*B*. *miyamotoi* antiserum for selection of survivor populations as shown in Fig 2B. Serial doubling dilutions of anti-*B*. *miyamotoi* mouse serum were made in 96-well plate starting with 1:5 dilution out to 1:80 in MKP-F media (50 μl). *B*. *miyamotoi* (1 x 10^5^ in 50 μl MKP-F) was added to each well (100 μl total volume). P8 culture was the seed culture for the parent controls incubated with either (equally diluted) normal mouse (BALB/c) serum (Innovative Research Inc., Novi, MI), or no serum. Sealed plates were incubated at 34° C for 18 hrs. Borrelial cell viability was checked by dark field microscopy daily. Contents of wells where no spirochetes were observed due to antibody killing were centrifuged for pellet collection, subcultured in fresh MKP-F (4 ml), and incubated at 34° C. Cultures were incubated for 2 weeks with periodic microscopic observation to determine growth for reisolates. Reisolates were culture expanded and stored at -80° C in 20% glycerol. The experiment was repeated using the same mouse antiserum and same parent (P8) grown from glycerol stock.

### Sodium dodecyl sulfate polyacrylamide gel electrophoresis (SDS-PAGE), immunoblot, recombinant Vmps

Recombinant proteins VlpC2 (ALU64348.1), VlpD9 (ALU64350), VlpD10 (ALU64352.1), VlpD1 (ALM31565), VlpD8 (ALU64349), and Vsp1 (AJA67245.2) were produced minus signal peptides in *E*. *coli* as described previously [24]. The proteins follow the naming scheme as listed by the accession numbers in the National Center for Biotechnology Information database (in parentheses). Recombinant proteins were electrophoresed on SDS-PAGE and silver stained (Pierce Silver Stain, Thermo Scientific, Rockford, IL) according to standard procedures and manufacturer’s directions. Proteins (100 ng/individual protein) were transferred to polyvinylidene fluoride membranes for immunoblotting according to standard procedures with mouse anti-*B*. *miyamotoi* (1:200), followed by incubation with alkaline phosphatase conjugated goat anti-mouse IgG (1:5000) with development by 5-bromo-4-chloro-3-indolyl phosphate / nitro blue tetrazolium.

### Nanopore and Illumina sequencing (NGS) and analysis

Amplicon DNA was sequenced using Oxford Nanopore Technologies’ SQK-NBD112.24 library preparation kit on an R10.4 flow cell using the Mk1B platform (following manufacturer’s protocol). The raw FAST5 and FASTQ sequencing data were submitted to GenBank (see Data Availability). The sequencing data were basecalled with the Guppy (v6.0.1) basecaller using the super accurate (sup) model. The basecalled data were demultiplexed with Guppy (v6.0.1) barcoder with the—detect_mid_strand_barcodes,—trim_barcodes, and—require_barcodes_both_ends parameters. For each demultiplexed dataset the data were filtered by length and quality using NanoFilt (v2.8.0) to select for reads greater than 900bp and q10 and less than 3kb.

The *vmp* genes were identified in both LB-2001 (GCF_017748095.1) and CT13-2396 (GCA_001767415.1) by classifying all potential transcripts in both genomes using InterProScan (v5.50–84.0) using the PFAM database [25]. The *vsp* genes contained the PF01441 motif and the *vlp* genes contained the PF00921 motif per Kuleshov and colleagues [26]. These transcripts were extracted from both genomes and combined into a *vsp* transcript dataset and *vlp* transcript dataset. Within both the *vsp* and *vlp* datasets it was possible that there would be redundant or nearly redundant transcript sequences, therefore to prevent issues during read mapping we clustered the transcripts in each dataset using cd-hit-est (v4.8.1) using the a percent sequence identity threshold of 95% (-c 0.95) to cluster the transcripts [27]. We assessed the presence of different *vmp* genes in the expression site by mapping the PCR amplicons to the different *vmp* transcript datasets. Because the amplicons may have both a *vsp* and a *vlp* present (as would be the case if a *vsp* gene is loaded in the expression site, see Fig 3) we first mapped the reads from each sample against the *vsp* transcript dataset using minimap2 (v2.22-r1101) (reads_mapped_to_vsp.sam) [28]. The reads that did not have a primary mapping to a *vsp* transcript were removed as a separate bam file for each sample (reads_unmapped_to_vsp.bam) using samtools view (v1.11) using the -Sbf 0x4 options [29]. The reads_unmapped_to_vsp.bam file was converted to a FASTQ file using samtools fastq and mapped to the *vlp* transcript dataset using minimap2 (reads_mapped_to_vlp.sam). The primary read mappings were filtered from the reads_mapped_to_vlp.sam file using samtools view with the -SbF 0x900 options (reads_mapped_to_vlp_primary.bam). Using samtools idxstats on the reads_mapped_to_vlp_primary.bam, we determined the number of reads mapping to each *vlp* gene for each sample. The primary mappings of the reads_mapped_to_vsp.sam file were filtered using samtools view with the -SbF 0x900 options and the same strategy using samtools idxstats employed for *vlp* genes was used here as well. With the strategy outlined above, we only considered primary read mappings to the different *vmp* genes when determining the profile of *vmp* genes found in the expression site for each sample.

Amplicon DNA was also sequenced by Illumina. Amplicon libraries from the PCR reactions were generated as previously described [30, 31] and sequencing was performed on the MiSeq system (Illumina, San Diego, CA) using MiSeq Reagent Kits Nano 500V2 according to the manufacturer’s protocol (Illumina). For sequence analysis, we used CLC Genomic Workbench (Qiagen) software, using reference generated from GenBank sequences of *B*. *miyamotoi* strain CT13-2396 (GCA_001767415.1) and LB-2001 (GCF_017748095.1). All reads that mapped to a reference sequence were further analyzed using MEGA7, and BLASTn software (https://blast.ncbi.nlm.nih.gov/Blast.cgi).

## Results

### Determination of the *vmp* within the expression locus by PCR

**Fig 1:**
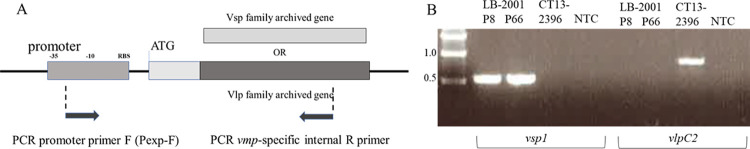
PCR of the expression locus. A) Illustration of the expression site with the active promoter consisting of the consensus ribosome binding site (RBS) and the -10 and -35 elements. ATG is the start codon of the coding sequence for the expressed Vmp, either a Vsp or a Vlp. The promoter specific forward primer (Pexp-F) is paired with a Vsp or Vlp specific internal reverse primer to amplify the gene being expressed for identification by amplicon sequencing. B) PCR amplification results of the *vsp1* and *vlpC2* genes in the expression site for strain LB-2001 low passage (P8) and high passage (P66), and low passage strain CT13-2396. NTC = no template control. M = molecular size markers that are denoted on left of panel.

The serotype of a *B*. *miyamotoi* population is determined by the expression of a Vmp-encoding gene that becomes situated in a single promoter-driven expression locus [17]. Therefore, we utilized PCR with a forward primer specific for the promoter region (Pexp-F) paired with reverse primers internal to Vmp pseudogenes to ascertain the gene being expressed in a population (Fig 1A).

The GenBank submissions for strains LB-2001 (GCA_000445425.4; GCF_017748095.1) and CT13-2396 (GCA_001767415.1) indicate that *vsp1* and *vlpC2* reside in the respective expression sites. (The CT13-2396 gene is annotated as *vlp5* and is an exact match for *vlpC2*). We generated reverse primers specific for *vsp1* and *vlpC2* to pair with Pexp-F for PCR to evaluate the presence of these genes. The *vsp1*-specific PCR produced an amplicon in both low passage (P8) and high passage (P66) strain LB-2001 indicating that *vsp1* remained in the expression site after multiple culture passages. As predicted, *vsp1* was not in the expression site for low passage CT13-2396 (Fig 1B). Conversely, the *vlpC2* primer set produced an amplicon in CT13-2396 but not LB-2001 (Fig 1B). Sequencing the amplicons verified their identity and confirmed the GenBank data for these strains. We used PCR to identify genes in the expression locus of a population in the subsequent experiments.

### Assessment of anti-*B*. *miyamotoi* mouse serum reactivity against Vmps

**Fig 2:**
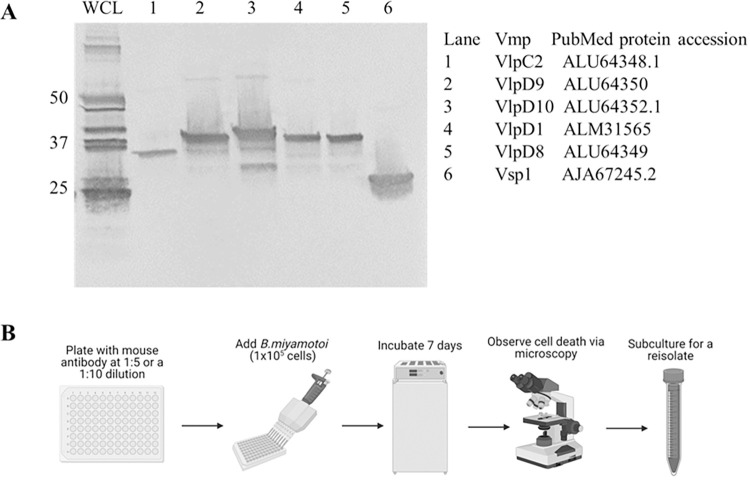
Polyclonal anti-*B*. *miyamotoi* reactivity and use in killing assay. A) IgG immunoblot of mouse polyclonal anti-*B*. *miyamotoi* LB-2001 antiserum against *B*. *miyamotoi* LB-2001 whole cell lysate (WCL) and recombinant Vlps. Molecular mass is indicated on the left by kDa. Recombinant proteins in lanes 1–6 are identified right of the panel. B) Step by step illustration of the antibody selection killing assay procedure.

We used previously reported mouse polyclonal anti-*B*. *miyamotoi* serum in the antibody selection killing assay [23]. The mouse antiserum was reactive against several *B*. *miyamotoi* proteins when immunoblotted against whole cell lysate, including those migrating around 37 kDa, the approximate size of Vlps, and at 25 kDa where Vsp1 migrates (Fig 2A, lane WCL). Furthermore, antibody reactivity against Vmps in the whole cell lysate was confirmed by blotting against recombinant Vlps and Vsp1 (Fig 2A, lanes 1–6). Vlps contain conserved regions that may exhibit cross-reactive epitopes, therefore antibodies represented in the polyclonal antiserum could have had the potential to eliminate multiple serotypes present in the parent population.

#### Antibody selection killing assays of *B*. *miyamotoi* and evaluation of the *vmps* in the expression site by PCR and DNA sequencing

Having clonal strains when conducting a genetic experiment is always optimal, however obtaining a *B*. *miyamotoi* clone has been problematic. We have been unsuccessful in generating a clone either by plating or serial dilution. We proceeded with the experiments without knowledge of the clonality of the parent stock culture we had obtained.

Low passage (P8) LB-2001 stock culture was grown to mid-logarithmic phase for the assay. Culture aliquots were incubated alone as a no serum control (NSC) or with mouse polyclonal anti-*B*. *miyamotoi* serum to apply selection pressure against the parent serotype population. The procedure for the *in vitro* killing selection is outlined in Fig 2B. After 7 days of antibody co-incubation in wells with dilutions at 1:5 or 1:10, we observed an absence of viable cells visible by dark field microscopy. Antiserum dilutions higher than 1:10 did not result in killing. We centrifuged the media from individual wells where live spirochetes were not observed, and resuspended pellets in fresh MKP-F followed by incubation and checked by microscopy daily for culture growth. Borrelial reisolate growth was observed at day 10, and by week 3 the culture achieved density for cell pellet collection. The experiment was repeated as a biological replicate using the same anti-*B*. *miyamotoi* antiserum against P8 culture. The results of each replicate experiment are described below as Replicate 1 and Replicate 2.

**Fig 3:**
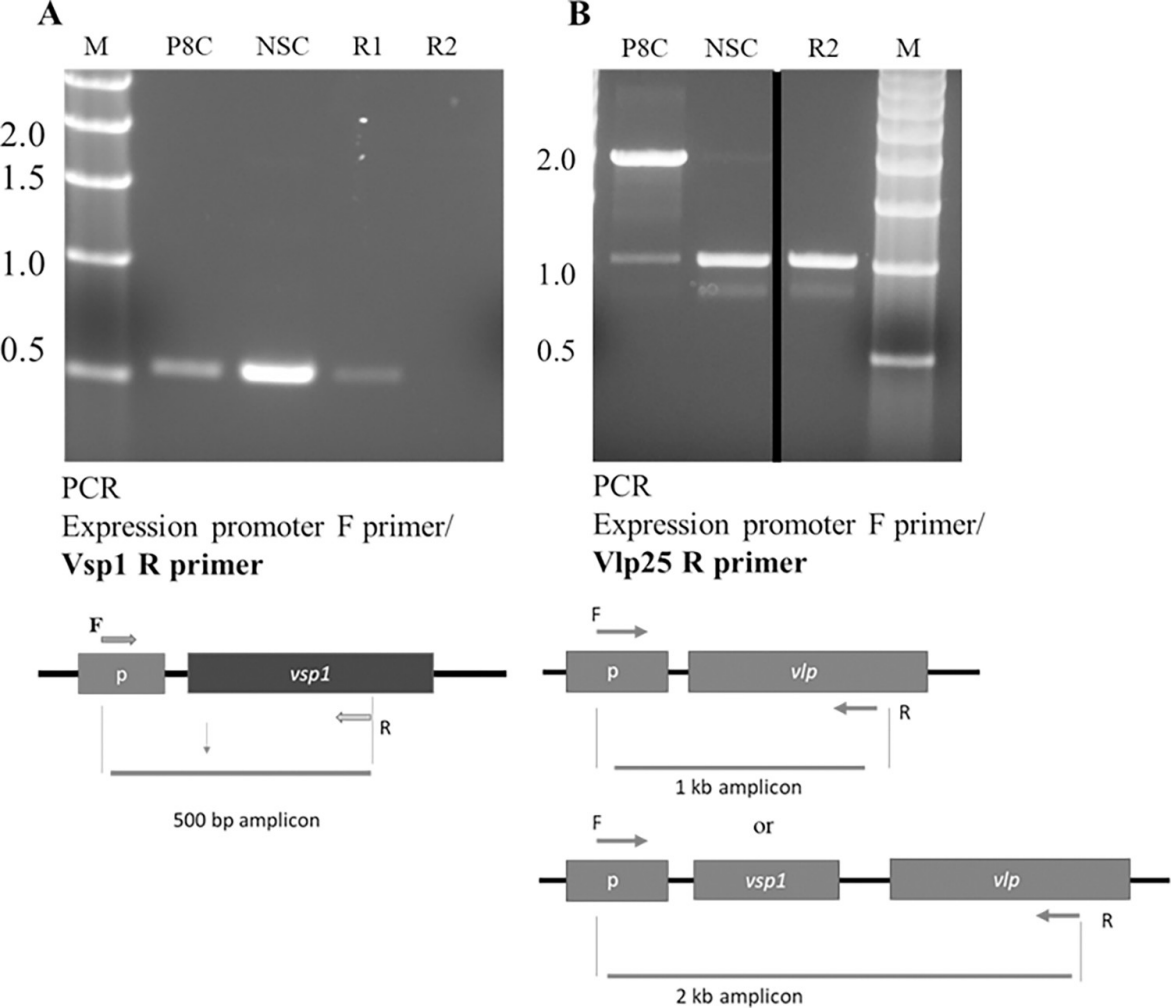
PCR of the expression locus for Replicate 1 parents and reisolates. A) Agarose gel of amplicons using the Vsp1 R primer with PCR illustration below the panel. B) Agarose gel of amplicons using the Vlp25R primer with PCR illustration below the panel. P8C = passage 8 parent stock culture used in the experiments; NSC = parent culture control incubated with no serum; R1 = reisolate 1; R2 = reisolate 2; M = molecular size markers that are denoted left of the panels in kilobase pairs. The black line in panel B indicates the spliced gel with removal of a lane. The juxtaposed lanes originated from the same original gel image.

In the first experiment (Replicate 1), PCR was performed using the Pexp-F and reverse *vsp1* (Vsp1R) primer pair on the parent controls and 2 reisolates that were recovered from antibody selection wells. A *vsp1* amplicon was detected and confirmed by DNA sequencing in the parent stock culture, parent incubation control, and reisolate R1 (Fig 3A lanes P8C, NSC, R1 respectively), whereas there was no amplification from reisolate R2 indicating that *vsp1* had been replaced by another gene (Fig 3A, lane R2).

Based on the nucleotide alignment of the 29 LB-2001 Vlp genes, we designed the Vlp25R primer from a conserved internal region of 25 Vlp pseudogenes of the delta and gamma subfamilies that shared sequence homology. PCR was performed on reisolate R2 with the Pexp-F and the Vlp25R primer pair generating an approximate 1 kb amplicon demonstrating that a Vlp gene was detectable within the expression locus (Fig 3B, lane R2). The P8 stock culture parent control produced 2 amplicons by PCR with the Pexp-F and Vlp25R primer pair (Fig 3B, lane P8C). The major 2 kb band represented a Vlp pseudogene located downstream from the expression site containing *vsp1*. However, a minor 1 kb band was also present which indicated a *vlp* in the expression site (see diagrams below panel Fig 3B). The no serum control parent amplicon pattern had changed from the P8 stock culture parent whereby the 2 kb amplicon was noticeably fainter and the 1 kb band more intense (Fig 3B, lane NSC). In addition, faint amplicons at approximately 0.9 kb were observed in NSC and R2 lanes. These observations suggested that subvariant(s) were present in these populations. Therefore, Illumina and Nanopore sequencing (NGS) of the 1 kb amplicons from the control parent (NSC) and reisolate (R2) were employed to determine their compositions by mapping to reference genomes LB-2001 (GCF_017748095.1) and CT13-2396 (GCA_001767415.1).

Sequencing results from the reisolate (R2) amplicon revealed multiple reads representing a profile of variants with the highest read counts matching Vlp genes bmLB2001_RS06360 and AXH25_RS05380 (CT13-2396 strain) suggesting these were the majority variants (S1 Fig). Conversely, the parent control had a predominant read identifying a match with a different Vlp gene from strain CT13-2396 (AXH25_RS05385). This gene has a 99% nucleotide match to LB-2001 *vlpD4* on lpD [protein id ALN43421.1]). Importantly, these results showed a dramatic change in predominant Vlp variant populations that arose in the reisolate differing from the parent following borrelial cell death from antibody selection. Moreover, we found the parent control culture was non-clonal by PCR and with low NGS read counts perhaps representing minority Vlp serotype populations in addition to the Vsp1 serotype (S1 Fig).

**Fig 4:**
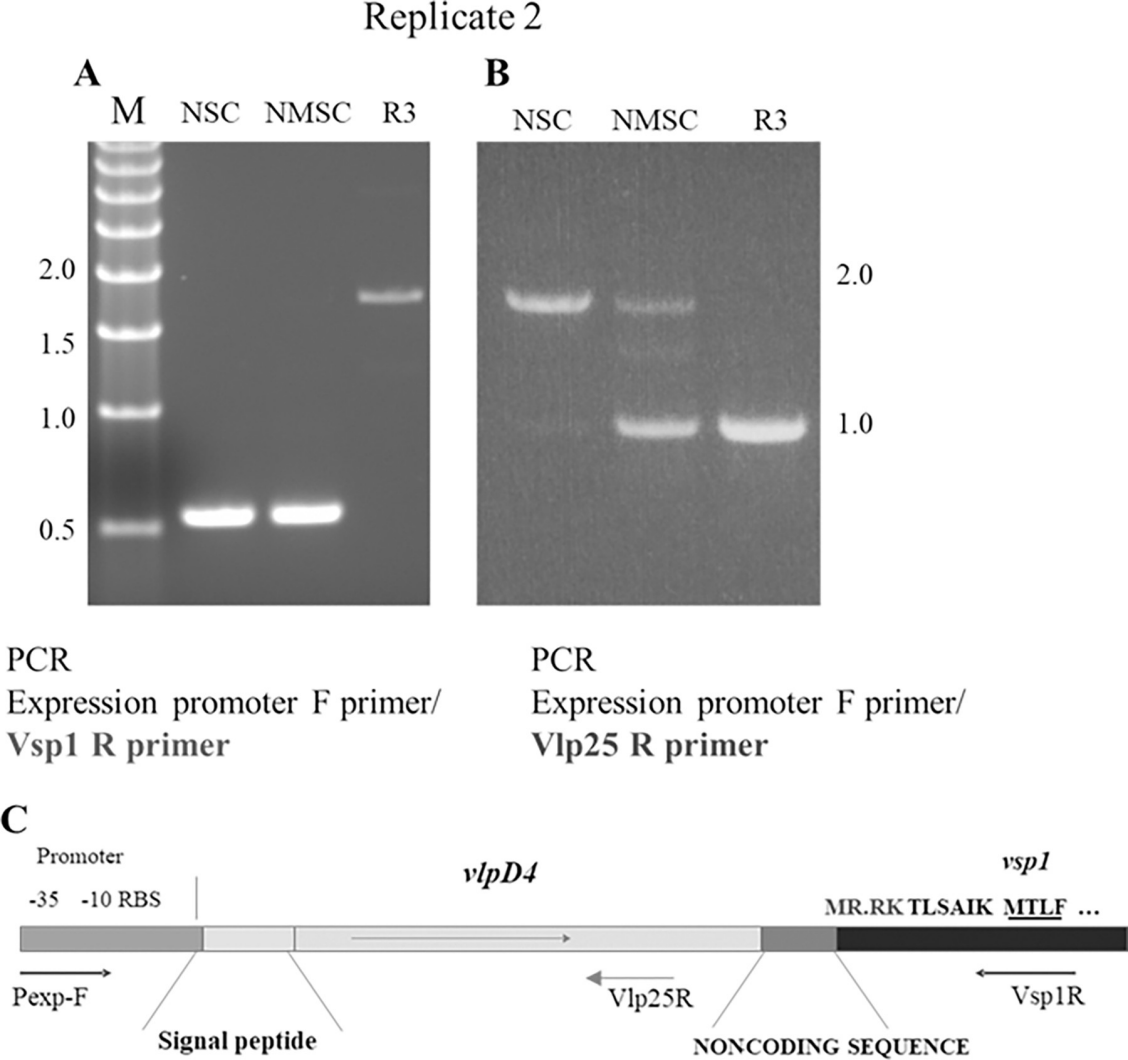
PCR of the expression locus for Replicate 2 parents and reisolate. A) Agarose gel of amplicons using the Vsp1R primer. B) Agarose gel of amplicons using the Vlp25R primer. C) Illustration of the expression locus of the reisolate R3 denoting *vlpD4* in the expression site with archived *vsp1* immediately downstream separated by a non-coding sequence. The genes are contiguous and located on lpD of strain LB-2001. Pexp-F forward primer and the Vsp1R and Vlp25R reverse primers are shown by arrows. The *vsp1* frameshift with a stop codon is shown with the resulting amino acid sequence change and a potential new translation start with a methionine is underlined. NSC = parent culture control incubated with no serum; NMSC = parent control culture incubated with normal mouse serum; R3 = reisolate 3. M = molecular size markers that are denoted left of the panels in kilobase pairs.

The antibody selection experiment was repeated (Replicate 2) under the same conditions, i.e. using the same P8 parent culture and polyclonal mouse antiserum as in Replicate 1. Antibody selection pressure from this experiment also produced a reisolate (R3). PCR analyses of the parent incubation controls (incubated with no serum or with normal mouse serum [commercially purchased BALB/c mouse serum–see Materials and Methods]) using the Pexp-F and Vsp1R primer pair revealed *vsp1* in the expression locus as expected (Fig 4A, lanes NSC and NMSC). However, the reisolate produced a 1.7 kb amplicon with this primer pair (Fig 4A, lane R3). Sanger sequence data of the amplicon revealed that a *vlp* gene had been translocated into the expression locus with a *vsp1* gene situated downstream accounting for the 1.7 kb size (Fig 4C). BLAST analysis identified the expressed *vlp* as strain LB-2001 *vlpD4* on plasmid lpD (GenBank protein accession ALN43421.1) with a non-coding *vsp1* positioned downstream from *vlpD4* on lpD (GenBank protein accession ALN43422.1). *vlpD4* has orthologs to strain CT13-2396 genes on lp41 and lp26 (Genbank accessions AXH25_RS05670 and AXH25_RS05385 respectively). The closest match to LB-2001 reference genome (submission GCA_017748085.1) was 87% identity on archived genes located on lp22 (bmLB2001_RS06455), lp31 (bmLB2001_RS05405), and lp38 (bmLB2001_RS05310).

PCR of the reisolate (R3) expression site using the Vlp25R primer produced an expected 1 kb amplicon representing a *vlp* as found from sequencing the 1.7 kb Vsp1R-generated amplicon (Fig 4B, lane R3), but NGS of this amplicon produced reads that mapped to other *vlps* in the reference genomes with a high read count for Vlp gene bmLB2001_RS06360 (S2 Fig). In the parent controls (Fig 4B, lanes NSC and NMSC), the presence of the approximately 2 kb amplicon band can be explained whereby *vsp1* is in the expression site with a Vlp gene downstream (refer to illustration in Fig 3B). However, like the parent control in Replicate 1, the 2 parent controls, each produced a 1 kb amplicon indicating a Vlp-expressing population(s) in addition to the Vsp1 serotype, thus representing a non-clonal population (Fig 4B, lanes NSC and NMSC). NGS of the 1 kb amplicons for each parent control revealed differences from each other in the number of reads, i.e., the NSC had low read counts of multiple genes, but the NMSC had high read counts for genes bmLB2001_RS06360 and AXH25_RS05385 (S2 Fig). The possibility exists that components in normal mouse serum may account for causing the variation seen between the NMSC and the control incubated without serum. The R3 reisolate also showed a relatively high read count for these two genes suggesting that these populations were not seriously affected by the antibodies and expanded as outgrowths from the parent.

Summarizing the results of Replicates 1 and 2, PCR data showed that i) *vsp1* was in the expression locus in parent controls but absent in reisolates R2 and R3; ii) the reisolates represented Vlp variants replacing Vsp1 as the dominant population; and iii) parent incubation controls were polyclonal possessing *vlps* in the expression site.

Other observations were notable. Of the 13 *vsp* pseudogenes in the LB-2001 genome, only *vsp1* (bmLB2001_RS0-5305 located on lp38) was found in the expression locus by NGS. A limited RNAseq analysis of a population from mouse blood in a previous investigation revealed a similar finding [17]. There are 29 *vlp* pseudogenes annotated in the LB-2001 genome (GCF_017748095.1) with subfamily representations designated as delta (n = 20), alpha (n = 4), gamma (n = 5), and beta (n = 0). All *vlp* pseudogenes that were identified by NGS in the parent and reisolates from both Replicates were of the delta subfamily with the alpha, beta, and gamma subfamilies not represented. We designed a separate Vlp reverse internal gene primer (Vlp4R) based on a conserved region from the nucleotide alignment of the alpha subfamily *vlp* pseudogenes. When paired with the Pexp-F primer, Vlp4R failed to amplify a Vlp alpha subfamily gene in the expression locus for any parent or reisolate.

**Fig 5:**
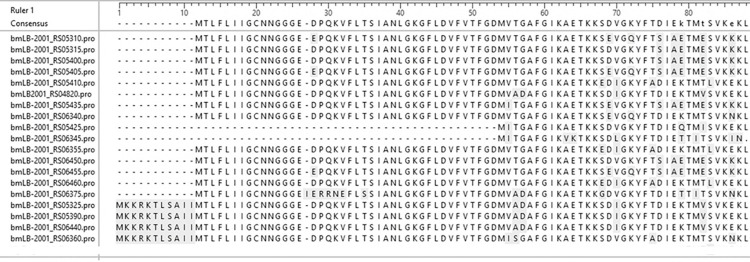
Amino acid alignment of the N-terminal coding sequence for the LB-2001 delta family Vlps as encoded by the archived genes. The proteins are annotated according to GenBank accession GCF_017748095.1. Shaded areas represent amino acid differences from consensus and illustrate the 4 genes that begin with MKKRKTLSAII and the beginning of divergence for individual pseudogenes translations following N-terminal conservation.

For Replicate 2 reisolate R3, sequencing data revealed that the coding sequence start for the *vlpD4* gene, when located in the expression locus, began with the amino acid translation of MKKRKTLSAII which is not encoded in most of the archived pseudogenes, but is present in 4 members of the delta Vlp subfamily (Fig 5). Vlp amino acid alignments revealed that most archived Vlp delta pseudogenes begin with a conserved motif of MTLFLIIGCNNGGGE extending for about 60 amino acids whereby heterogeneity defining individual Vmp genes proceeds. The genes that code for MKKRKTLSAII may be an indicator for genes that have a higher frequency of recombination into the expression site perhaps by the presence of non-coding upstream homology sequences [12, 15] and should be the subject of further study.

### Protein analysis of parents and reisolates

**Fig 6:**
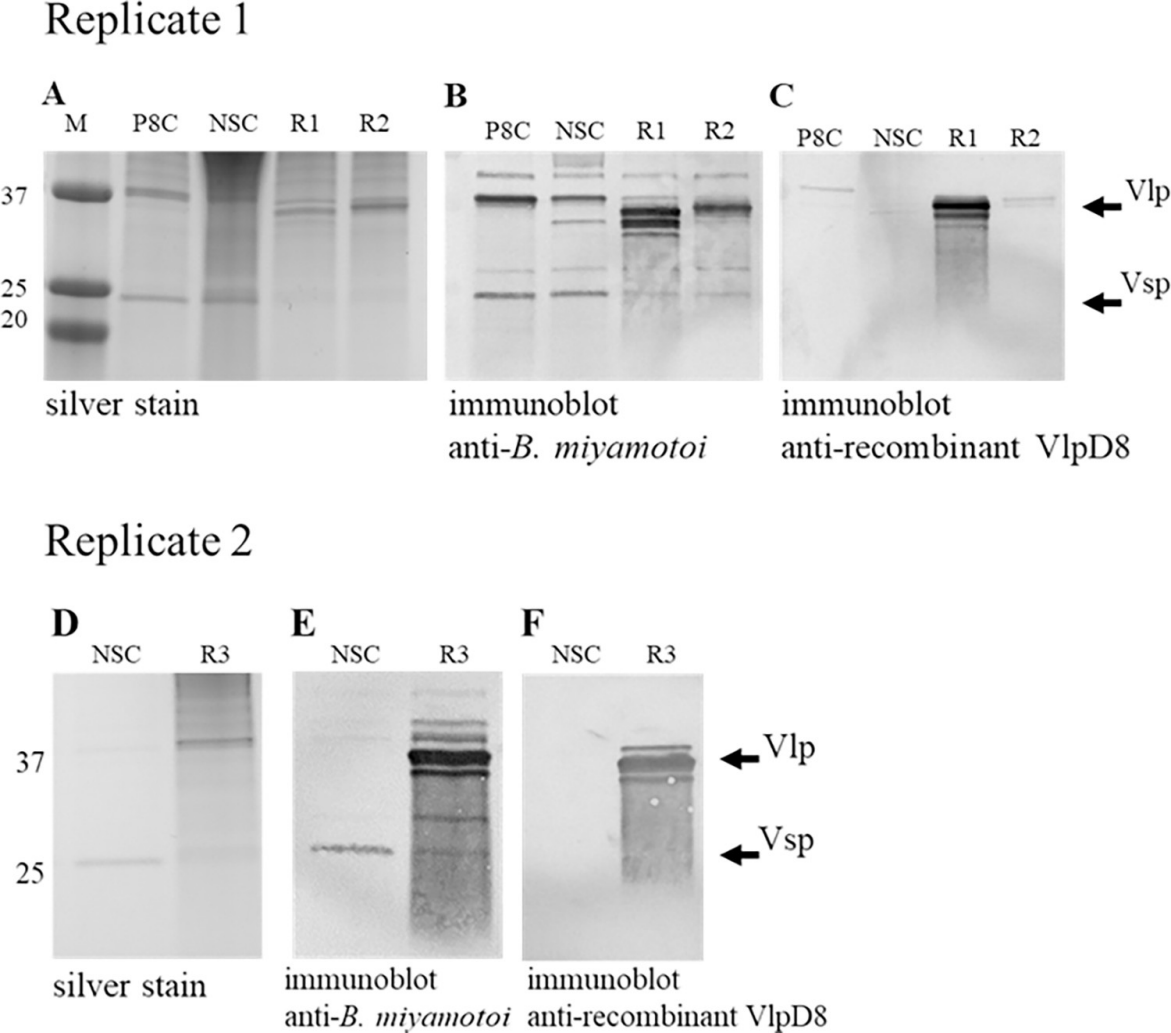
Vmp parent and reisolate comparisons by SDS-PAGE and immunoblot. A) Replicate 1 SDS-PAGE silver stained; B) Replicate 1 immunoblot with mouse anti-*B*. *miyamotoi* antiserum; C) Replicate 1 immunoblot with mouse anti-recombinant VlpD8 antiserum; D) Replicate 2 SDS-PAGE silver stained; E) Replicate 2 immunoblot with mouse anti-*B*. *miyamotoi* antiserum; F) Replicate 2 immunoblot with mouse anti-recombinant VlpD8 antiserum. P8C = passage 8 parent stock culture control; NSC = parent culture control incubated with no serum; R1 = reisolate 1; R2 = reisolate 2; R3 = reisolate 3. M = molecular size markers that are denoted left and right of the panels in kDa. Arrows on the right of the panels designate molecular migration for Vlp and Vsp bands.

Protein profiles of the Replicate 1 and 2 parent controls and reisolates were compared by SDS-PAGE of whole cell lysates. Gel silver staining showed differences in protein bands around 37 kDa and 25 kDa where the Vlps and Vsps migrate respectively (Fig 6A and 6D). Divergent Vmp profiles between parents and reisolates became clear after immunoblotting with mouse anti-*B*. *miyamotoi* serum (Fig 6B and 6E). Vsp1, representative of the predominant serotype in the parents, was diminished or absent in the reisolates and was replaced by Vlp serotypes. We repeated the immunoblots using mouse anti-recombinant VlpD8 antiserum and verified that the immunoreactive bands seen in the anti-whole cell blots were indeed Vlps (Fig 6C and 6F). There is broad homogeneity between Vlps, therefore antigenic cross-reactivity was to be expected from blotting with the anti-recombinant VlpD8. However, it is notable that the two Replicate 1 reisolates were expressing different Vlps as shown by the absence of reactivity against anti-recombinant VlpD8 (Fig 6C). These results provide additional evidence for the changes in the variant populations following the antibody selection assay as seen by the PCR and sequencing data.

## Discussion

Recognition and mechanisms of antigenic variation by gene conversion in *B*. *hermsii* and *B*. *turicatae* have been well studied and documented for over 40 years [5, 6, 18, 32–34]. Intrigued by recent findings regarding another member of the RF spirochetes, *B*. *miyamotoi*, we proposed an initial more comprehensive look at this phenomenon. Previous sequence analysis of selected plasmids of strain LB-2001 provided a description of multiple and diverse *vmps* demonstrating that *B*. *miyamotoi* possessed the genetic makeup capable of serotype switching [17]. Wagemakers et al provided the first in vitro demonstration of gene conversion in *B*. *miyamotoi* LB-2001 by a monospecific anti-Vsp1 antibody-mediated selection that killed the majority population resulting in a new population expressing *vlpC2* [19]. Our experiment was a modification of the Wagemakers et al study whereby we used a polyclonal anti-*B*. *miyamotoi* antiserum to mediate selection on the parent population with subsequent PCR and DNA sequence identifications of the expression locus gene. Armed with antibodies against multiple Vmps in the killing selection assay, we hypothesized that the low passage parent strain would produce new serotypes by antigenic variation to evade the antibody attack.

Genomic sequencing data of the LB-2001 strain from the Barbour (GCA_000445425.4) and Kneubehl et al (GCF_017748095.1) GenBank entries showed that *vsp1* was situated in the expression locus. Accordingly, Vsp1 was shown to be the dominant antigen expressed by LB-2001 in the Wagemakers et al study [19]. Likewise in our study *vsp1* was present in the expression locus of our parent strain suggesting it was the dominant serotype in the stock culture that was used as the initial parent isolate. This contrasted to the North American strain CT13-2396 that had a different Vmp gene in the expression site, an indication of subtle, yet distinctive, genetic rearrangements that have been reported to occur among *B*. *miyamotoi* strains in close geographic proximities in the United States [31].

Variants were reisolated from the antibody selection cultures demonstrating survival or evasion of the polyclonal antibody killing pressure by a population(s). Subsequent PCR analyses of the reisolated variants demonstrated that in the absence of or addition to *vsp1* different *vlp* pseudogenes were detected in the expression locus. NGS and Sanger sequencing demonstrated that the single PCR band observed by gel electrophoresis was comprised of multiple amplicons indistinguishable by size on agarose gels indicating a dominant population but also other minority variant populations that emerged from the antibody selection experiment. This finding was confirmed by protein analyses demonstrating multiple seroreactive Vlp bands in the lysate immunoblots.

**Fig 7:**
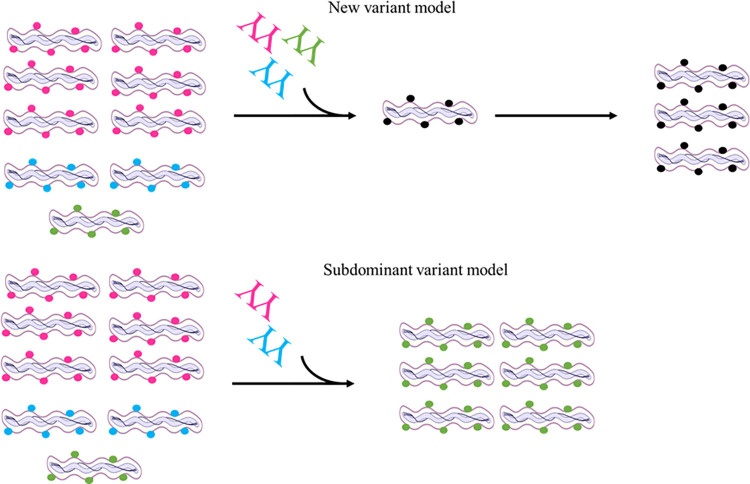
Hypothesized models for *B*. *miyamotoi* antigenic variation. The new variant model proposes that anti-Vmp antibodies eliminate the corresponding serotypes expressing those Vmps resulting in antigenic switching by gene conversion to generate a new Vmp serotype population. The subdominant variant model proposes that anti-Vmp antibodies eliminate the corresponding serotypes expressing those Vmps, but has no effect on a variant population that subsequently expands.

We also saw evidence suggesting that serotype populations could arise by continued culture growth absent of selection pressure as seen by changes in the parent strains used in our experiments. The Vsp1 serotype was the majority population in the parents but NGS and PCR analyses revealed profiles of subvariant Vlp-expressing populations. This finding indicated that low passage stock parent cultures, although with an apparent Vsp1 majority serotype, were not clonal but also were comprised of minority Vlp populations, thus introducing two hypotheses for the emergence of the variant serotypes following antibody selection (Fig 7). For the new variant model, polyclonal anti-Vmp antibodies would eliminate spirochetes of corresponding serotypes allowing gene conversion to generate new variants resistant to the antibodies. A subdominant variant model would entail a serotype present in the population unaffected by borrelicidal antibodies that expands following elimination of other serotypes present. Our findings here support the subdominant variant model and additional studies are needed to ascertain whether *B*. *miyamotoi* is capable of antigenic switching from a clonal population by the new variant model as described for *B*. *hermsii*. The fundamental mechanisms that trigger antigenic variation in response to antibody binding to Vmps or recognition of antibody killing by the borrelial cell have not been explored. Studies in this area are worthy of future research consideration.

Cultures existing as multiple serotype populations are not unique to *B*. *miyamotoi*. Ras et al reported that antigenic variation occurred spontaneously in *B*. *turicatae* during in vitro cultivation without antibody mediated selection [35]. Low frequency *B*. *hermsii* variants have been detected during expansion from either culture or animal infections. The antigenic switches occurred spontaneously and therefore it was inevitable for new serotypes to appear in a population after expansion of more than 100–1000 cells [5, 36, 37].

This study also demonstrated that although we used the same parent strain stocks and polyclonal antibody in the killing assays, reproducibility in producing serotypes was not assured. Sequencing also revealed gene sequences that did not match completely with archived Vlp genes in the current complete LB-2001 assembly (GCF_017748095.1) although there were matches found in the assembly for strain CT13-2396. The LB-2001 genome NGS read data did not indicate missing genes from the assembly. This observation may indicate intragenic recombination occurring prior to *vlp* translocation to the expression site as has been described for *B*. *hermsii* [11, 38]. Additionally, segmental gene conversion may occur within the expression locus. Another possibility is that parent cultures are not static but are in rearrangement flux. For example, Sanger DNA sequencing of the expression site of the Replicate 2 reisolate identified a *vmp* that had replaced *vsp1* as *vlpD4* from the Barbour LB-2001 assembly submission (GCA_000445425.4) and to 2 genes from strain CT13-2396. However, there was not an exact nucleotide match for this gene in the Kneubehl et al LB-2001 assembly submission (GCF_017748095.1), the closest being 87% for a few silent genes. An explanation for the discrepancy may be that a *vlpD4* homolog was not present in that reference genome suggesting that DNA prepared for genome sequencing may depend on the context of culture passage/history of the isolate. Therefore, a genome sequence generated from a strain may be considered a “snapshot” of the population from which the DNA is purified.

An alternate explanation for multiple variants may be the presence of an additional expression locus. A BLAST search with the expression promoter sequence located on the LB-2001 lp38 (CP072482.1) and lpB (CP010328.2) revealed a match on lpC (KR919748.1) from the Barbour assembly submission (GCA_000445425.4), however this was not seen in the Kneubehl et al assembly (GCF_017748095.1) or the CT13-2396 (GCA_001767415.1) GenBank submissions. Although it seems unlikely for more than a single unique expression site, further investigation should be considered.

The mouse polyclonal antiserum used in the killing experiment was generated from a needle-inoculation infection of a low passage *B*. *miyamotoi* LB-2001. The immunoblot profile of this antiserum against *B*. *miyamotoi* whole cell lysate was reactive to Vsp1 and bands in the 37 kDa area where Vlps migrate. This observation signifies that either the low passage isolate used to inoculate the mouse was composed of multiple serotypes, or the mouse elicited antibodies against new variants during the infection time frame, or both. This result agrees with an immunoproteomic study from our laboratory whereby several Vlps were identified as immunogenic by needle- or tick-infected mice [24]. A similar finding was reported by Lopez, et al whereby these investigators described multiple immunogenic *B*. *hermsii* Vmps in an immunoproteomic study [39]. Having clonal strains when conducting a genetic experiment is always optimal, however obtaining a *B*. *miyamotoi* clone has been problematic. We have been unsuccessful in generating a clone either by plating or serial dilution. Our preliminary work in this area has suggested that *B*. *miyamotoi* requires a certain cell density for expansion, therefore obtaining a clonal population from a single cell may be challenging.

In this initial look at the phenomenon of *B*. *miyamotoi* antigenic variation, we had the limitation of the number of replicates performed and we were not able to truly quantify the different Vmp populations loaded in the expression site solely by the NGS read numbers. Our data should be interpreted as a profile characterization of multiple expressed Vmp populations. Further studies will be required to determine the exact number of Vlp variants in a population and the frequency of individual *vmp* gene conversions.

In conclusion, our observations of borrelial cell death by exposure to anti-*B*. *miyamotoi* antibodies demonstrates that *B*. *miyamotoi* antigenically shifts in response to selection pressure. However, the possibility remains whether our findings of reisolated new variant populations are the result of i) gene conversion in response to antibody killing, or ii) the expansion of minority populations resistant to anti-Vmp antibodies, or iii) both. We have also corroborated the results reported by Wagemakers et al who demonstrated gene conversion *in vitro* when cells were subjected to anti-Vsp1 antibodies. This study was designed to provide initial observations and determination of the extent and possibilities of *B*. *miyamotoi* antigenic switching when subjected to antibody killing selection pressure. Validation and determination of the type, number, and frequency of serotype variants that arise during in vivo animal infections remains to be performed preferably by utilizing infected tick challenge. Our investigation and that of others clearly show that *B*. *miyamotoi* can switch antigenically *in vitro*, but how this relates to human infections where relapsing fever presentation is a minority of clinical cases described in the literature is an important question to be answered.

## Supporting information

S1 Fig Bar graph designating number reads per gene by Nanopore NGS for the Replicate 1 no serum control (NSC) parent and reisolate R2. (TIF)

S2 Fig Bar graph designating number reads per gene by Nanopore NGS for the Replicate 2 no serum control (NSC) parent, the normal mouse serum control (NMSC) parent, and reisolate R3. (TIF)

S1 Raw images (PDF)
