# Supplementary figures and images for: Analysis of variable major protein antigenic variation in the relapsing fever spirochete, Borrelia miyamotoi, in response to polyclonal antibody selection pressure
Source: PLoS One. 2023 Feb 24;18(2):e0281942. doi: 10.1371/journal.pone.0281942 (PMC9955969; doi:10.1371/journal.pone.0281942)

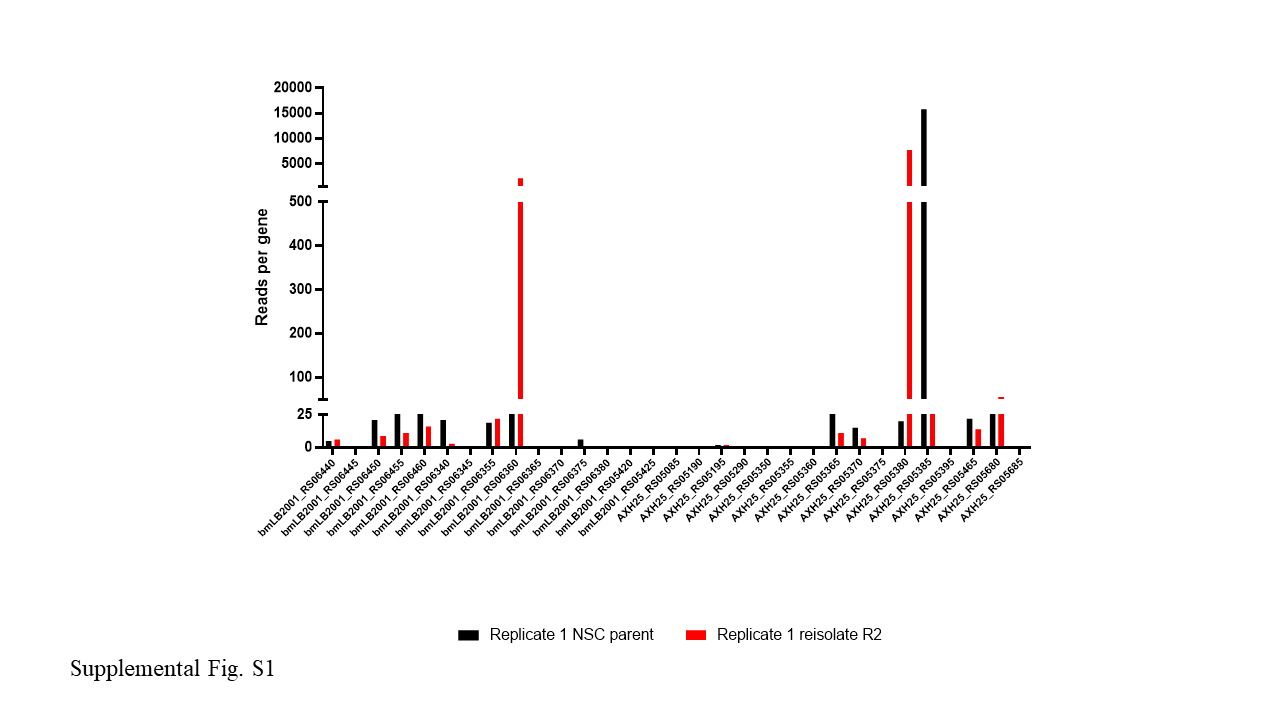

Supplement: S1 Fig — (TIF) [file pone.0281942.s001.tif]

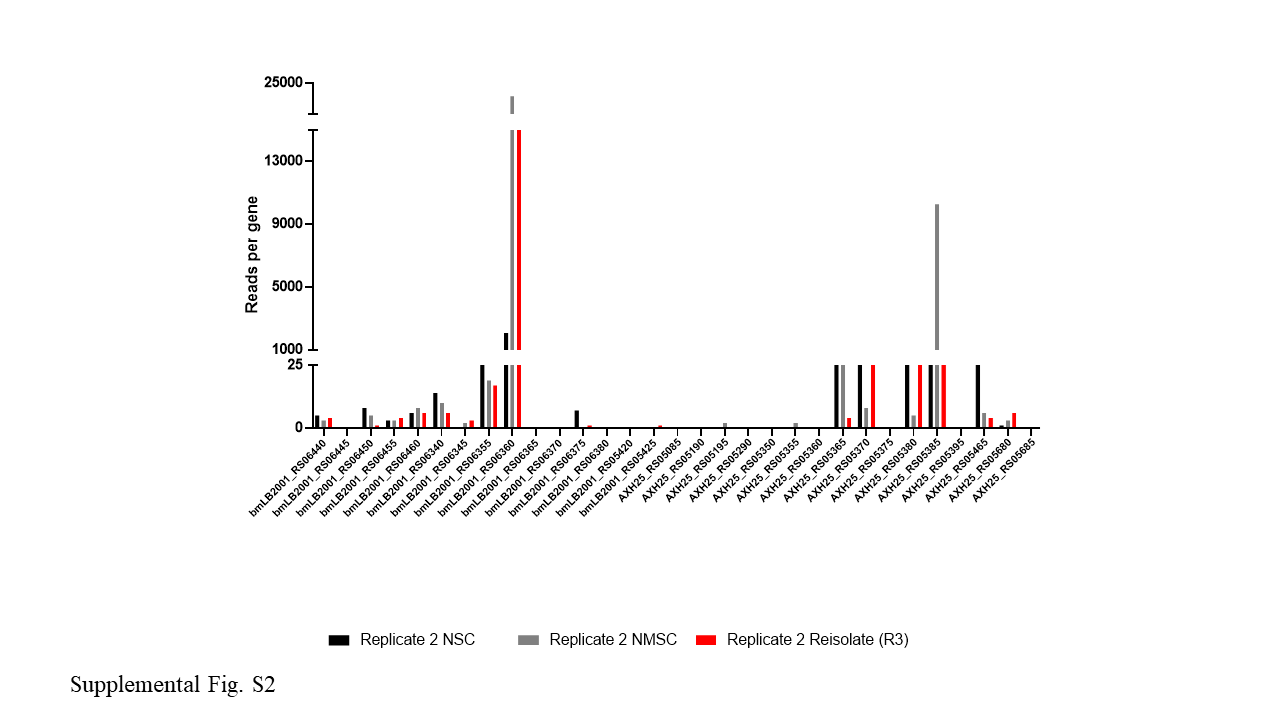

Supplement: S2 Fig — (TIF) [file pone.0281942.s002.tif]

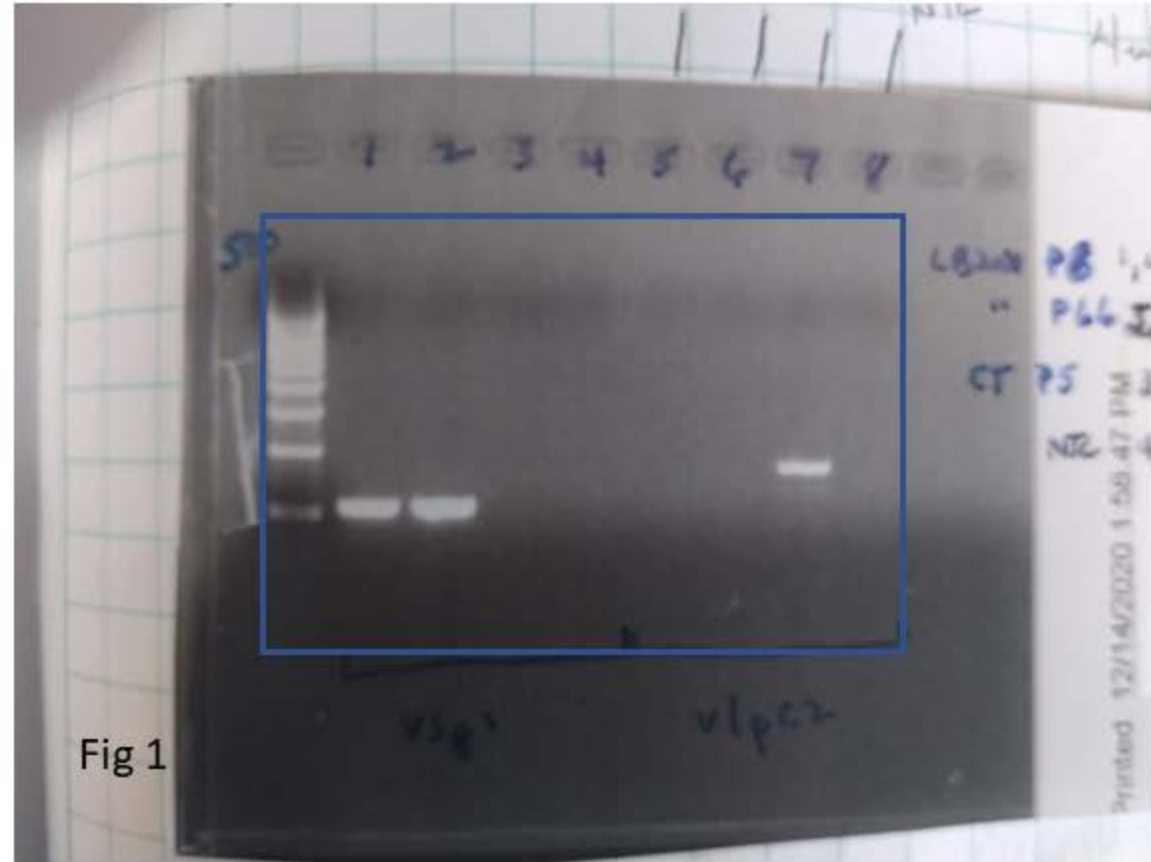

Fig 1

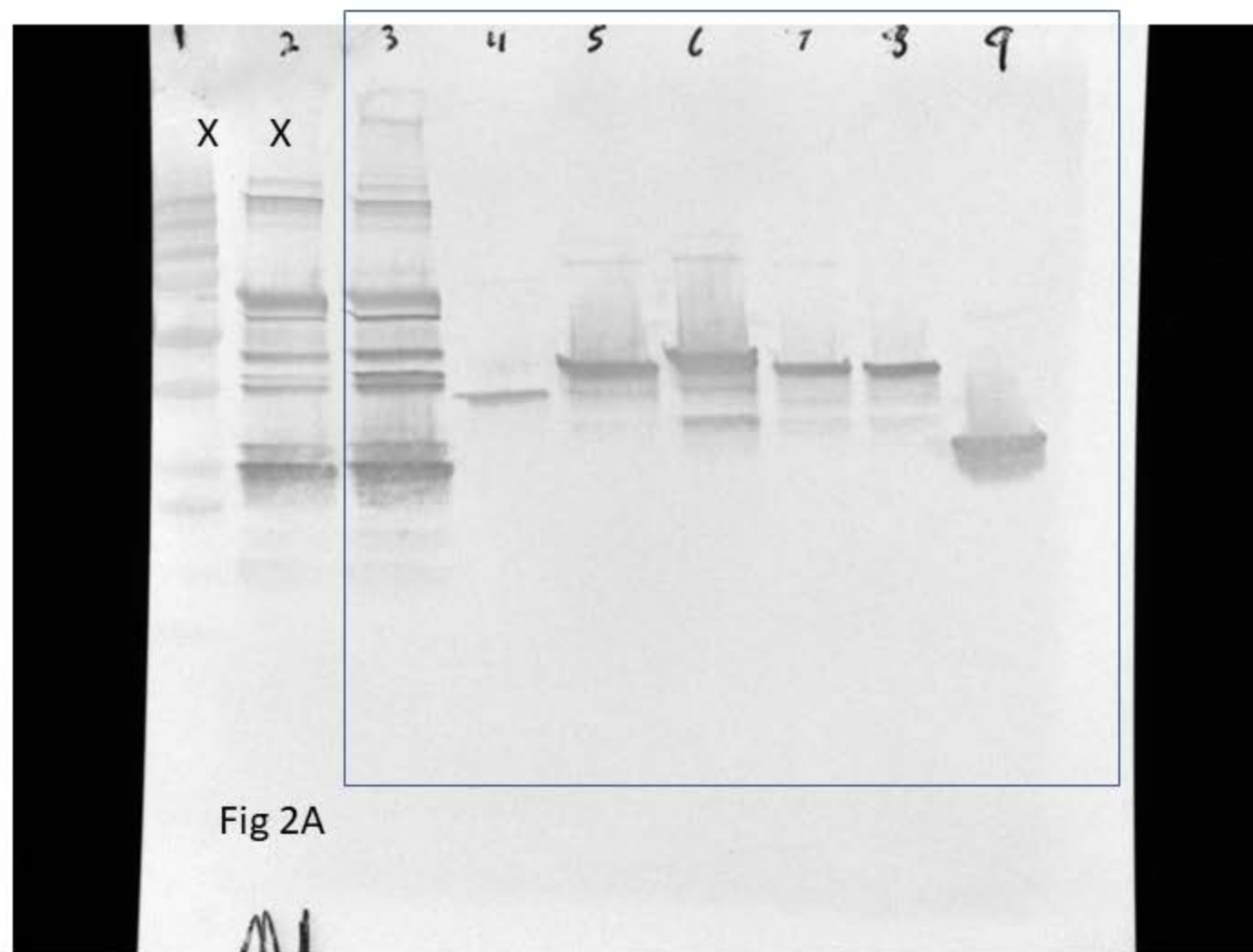

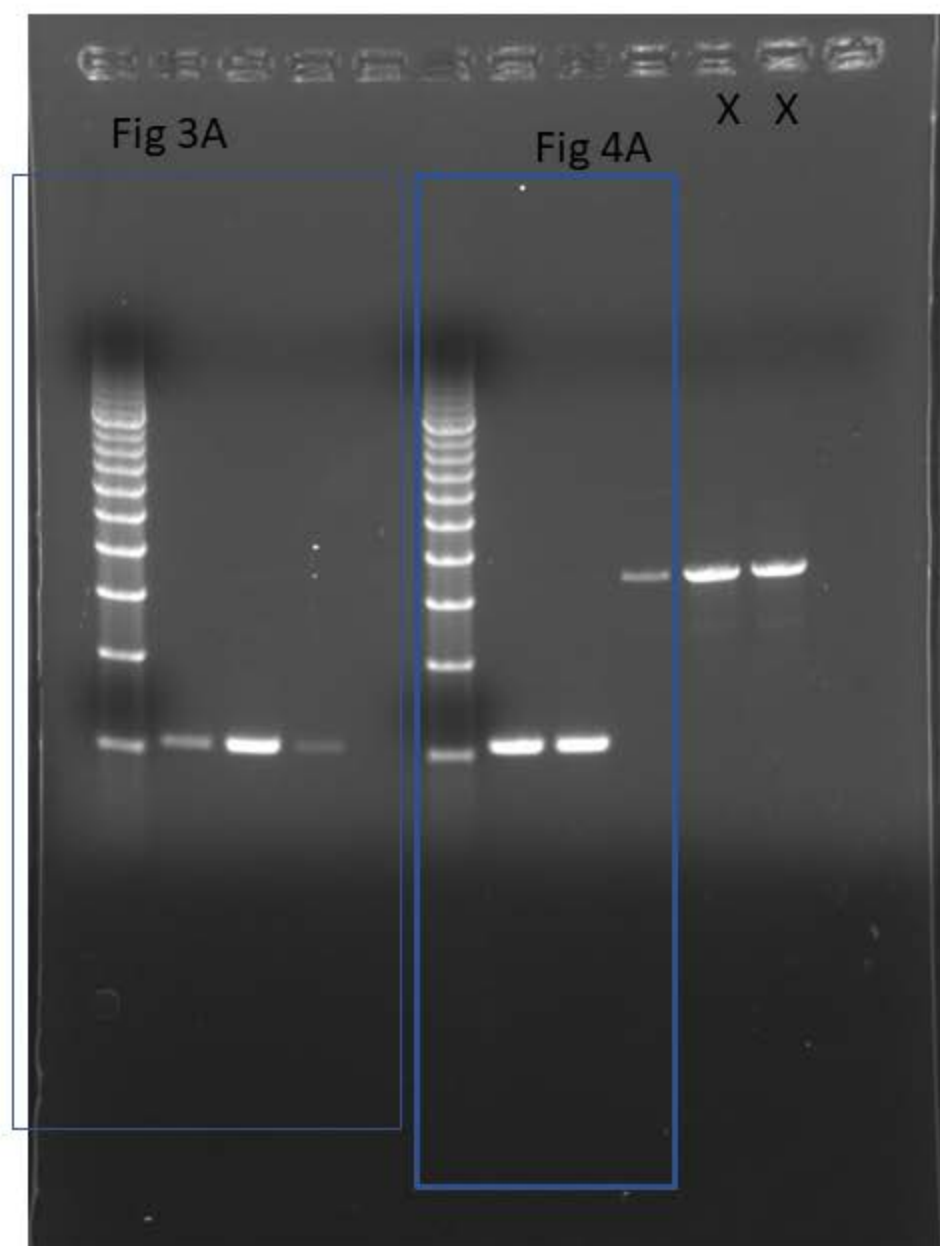

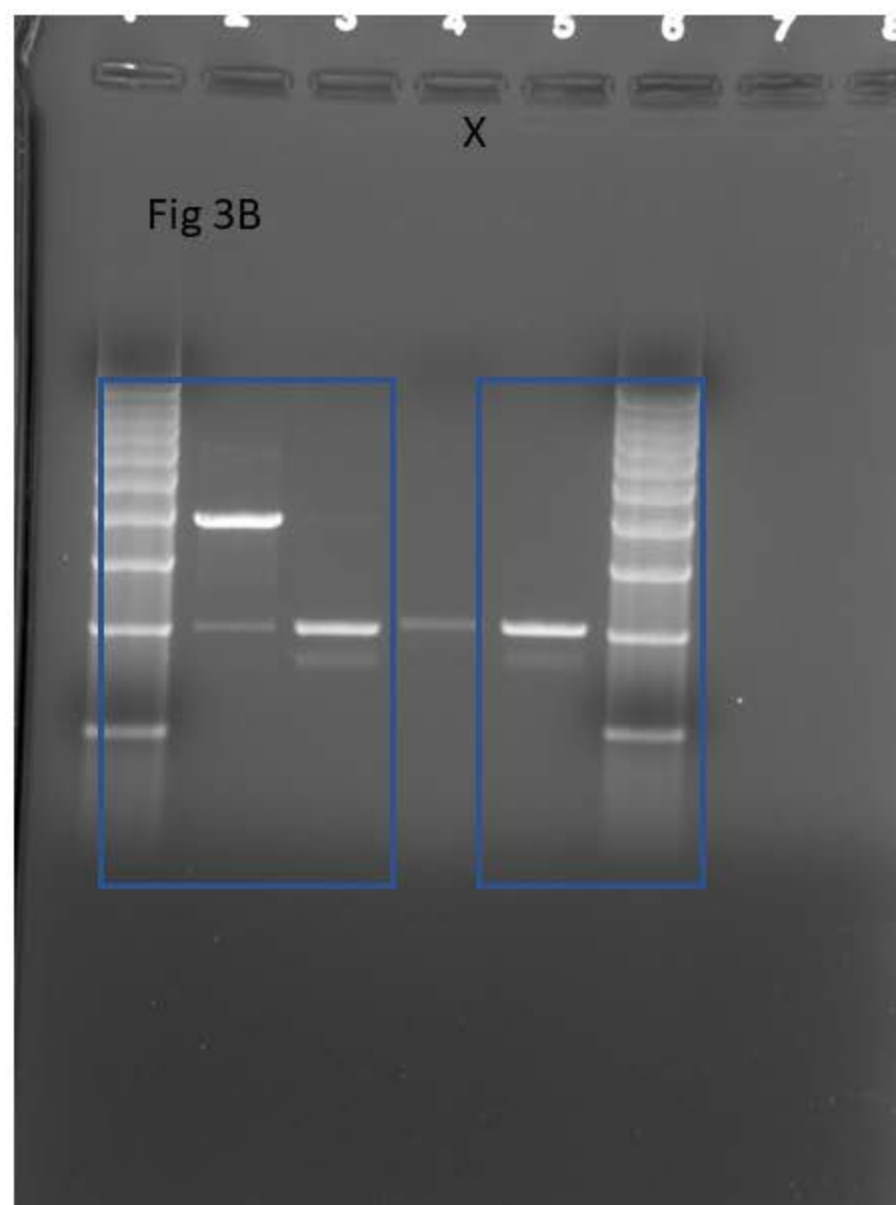

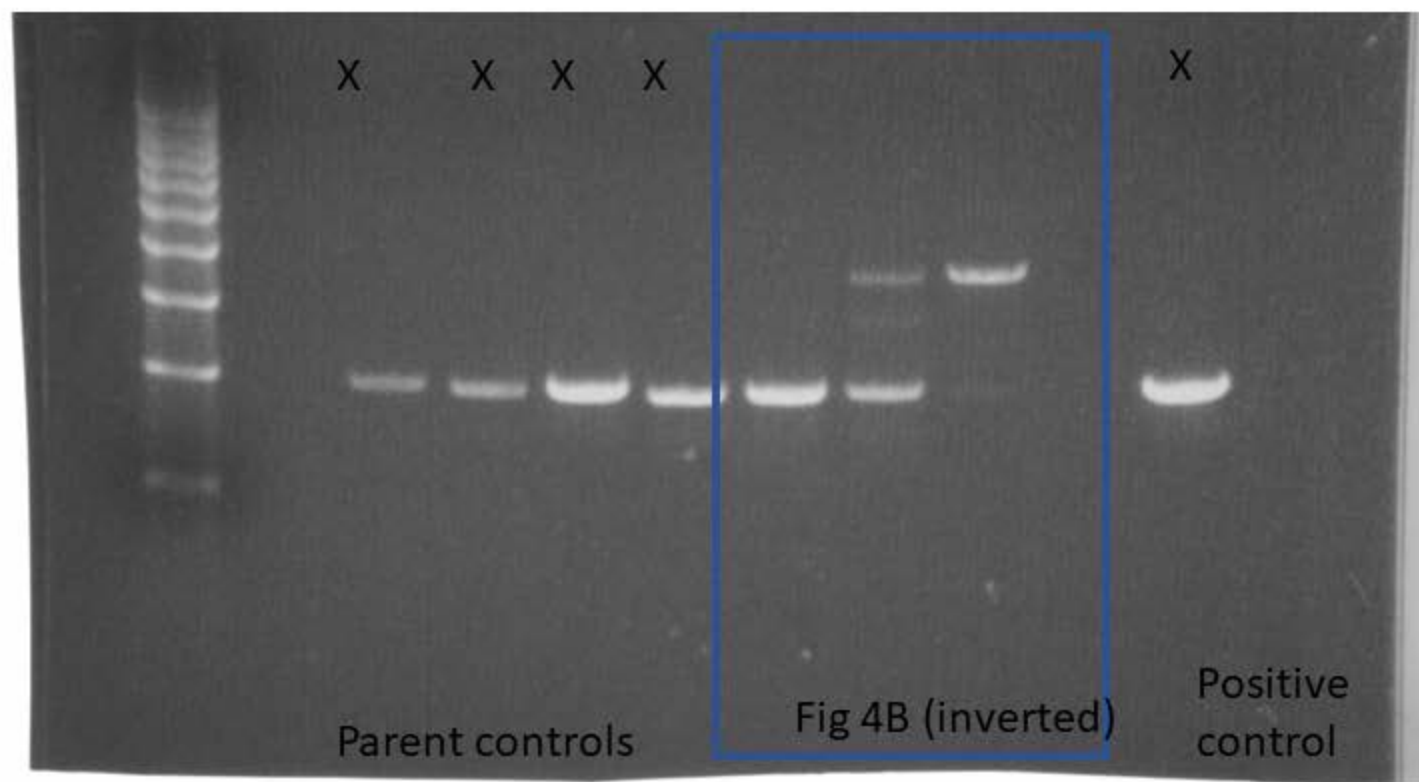

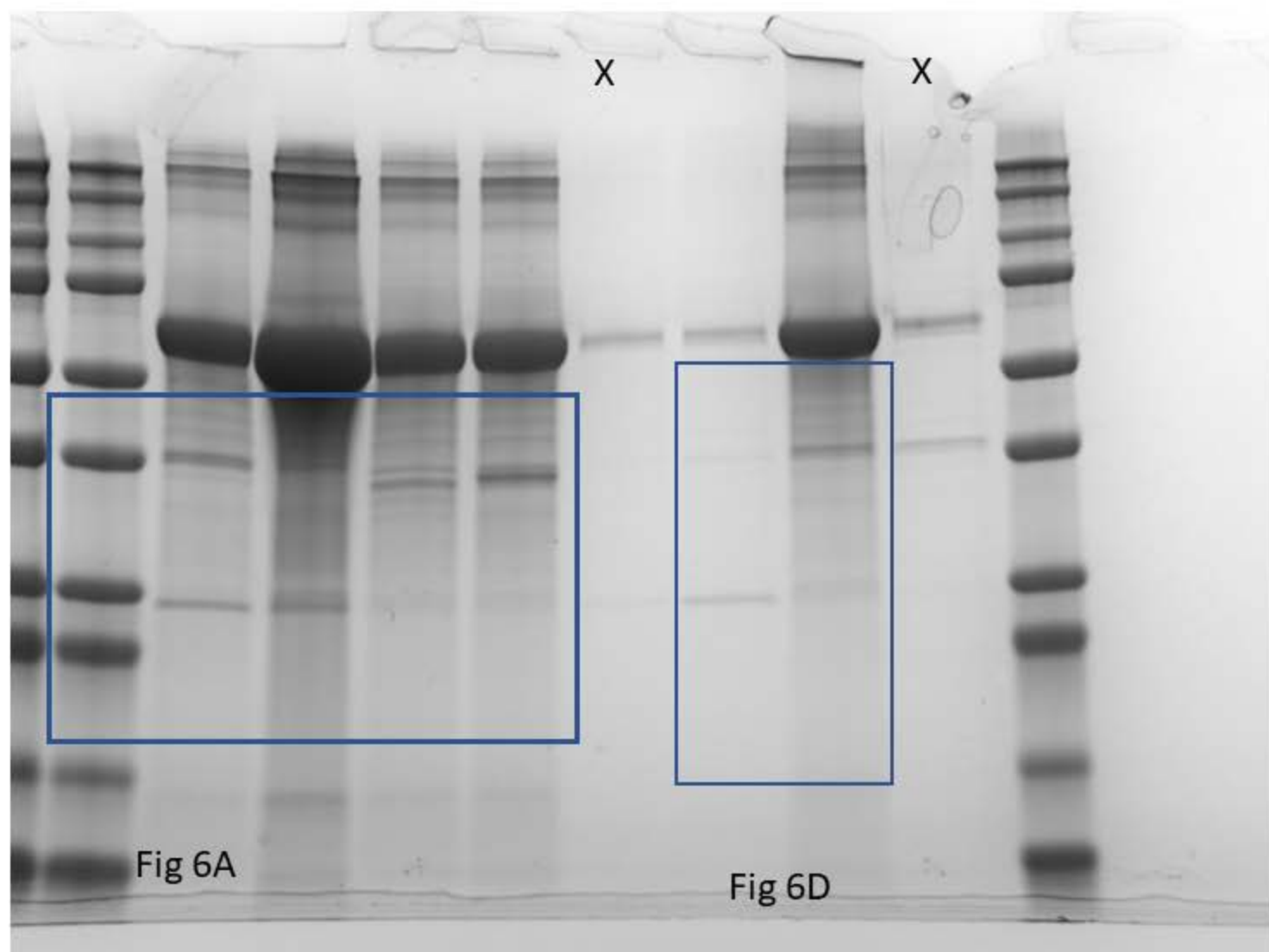

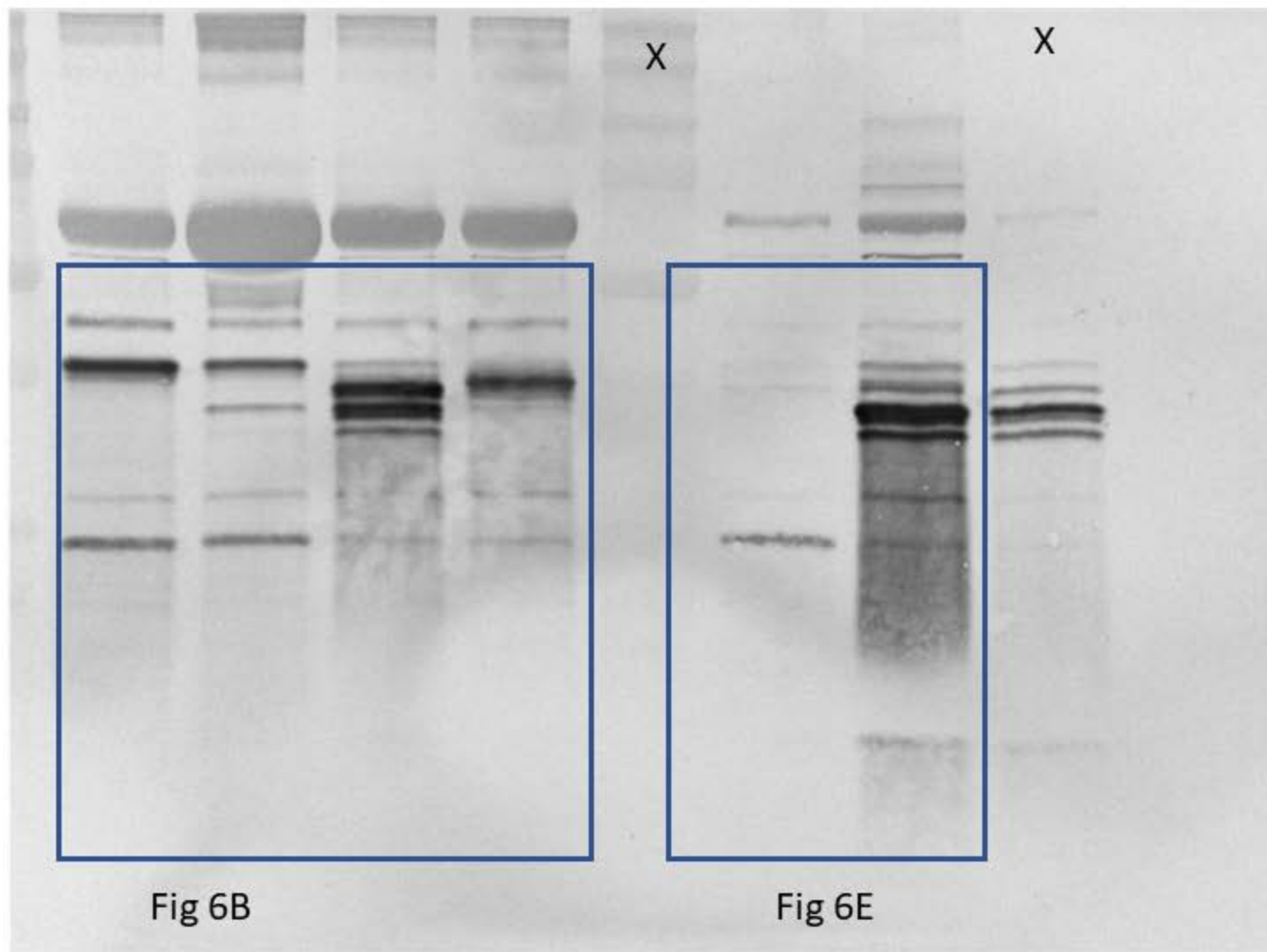

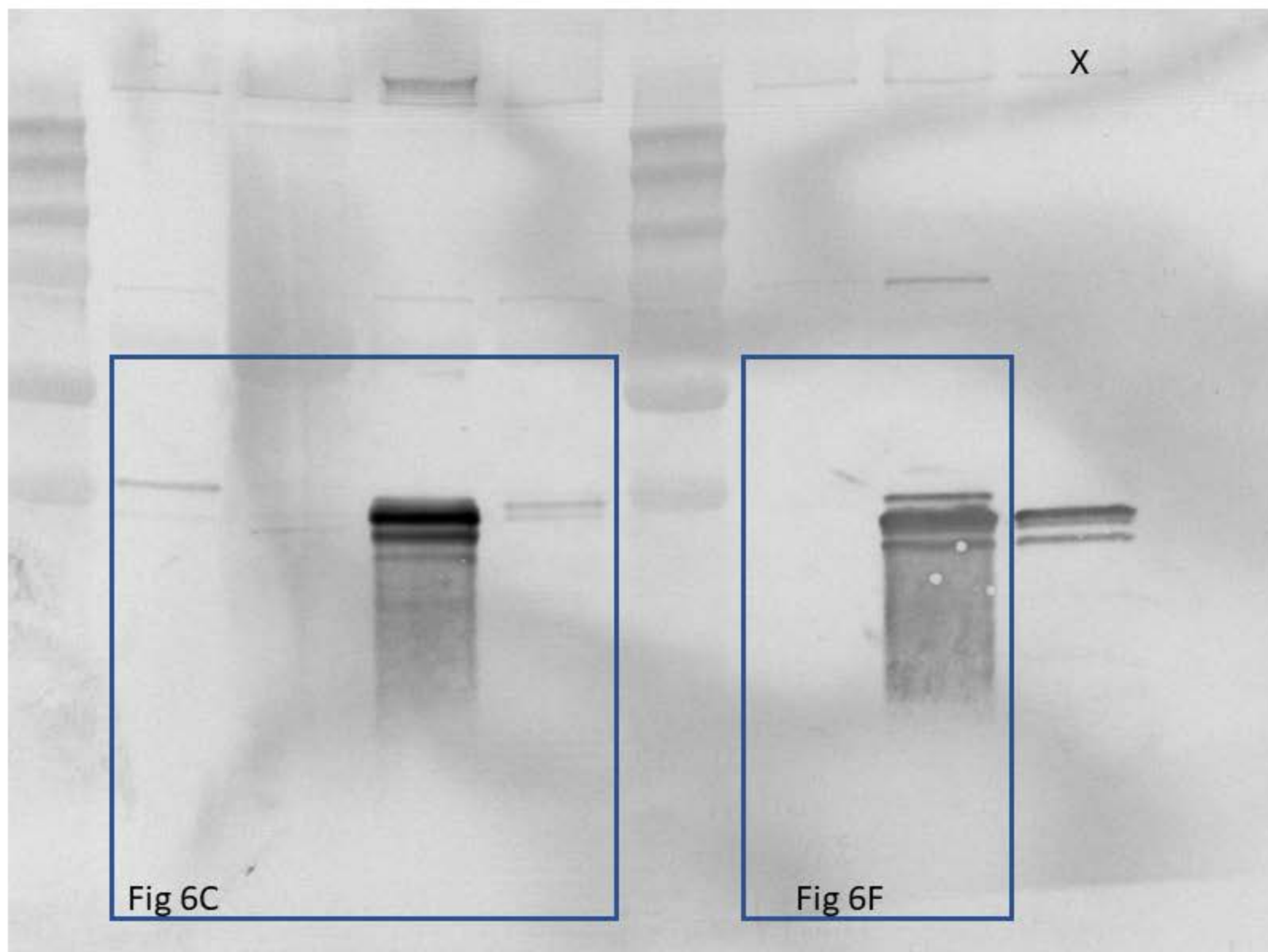

Supplement: S1 Raw images — (PDF) [file pone.0281942.s003.pdf]
